# Novel Biomarkers: Soluble Urokinase-Type Plasminogen Activator Receptor and Procalcitonin- and Histological Chorioamnionitis after Preterm Premature Rupture of Membranes

**DOI:** 10.1007/s43032-024-01678-6

**Published:** 2024-09-03

**Authors:** Kati Jalkanen, Anita Virtanen, Janne Aittoniemi, Heidi Flinck, Sinikka Ampuja, Heini Huhtala, Kati Tihtonen

**Affiliations:** 1 Department of Obstetric and Gynecology, Tampere University Hospital, Elämänaukio, Kuntokatu 2, Tampere, 33520 Finland; 2 Department of Clinical Microbiology, Fimlab Laboratories, Tampere, Finland; 3 Department of Pathology, Tampere University Hospital, Tampere, Finland; 4 Faculty of Social Sciences, Tampere University, Tampere, Finland

**Keywords:** Soluble urokinase-type plasminogen activator receptor, Procalcitonin, Amniotic fluid, Preterm premature rupture of membranes, Histological chorioamnionitis

## Abstract

Fetal inflammatory response syndrome or infection after preterm premature rupture of membranes (PPROM) increases neonatal morbidity in preterm deliveries. Biochemical markers from the amniotic fluid (AF) have been used to evaluate possible intra-amniotic infection during the asymptomatic phase after PPROM. This study aimed to describe whether soluble urokinase-type plasminogen activator receptor (suPAR) or procalcitonin (PCT) from AF or maternal sera could reveal fetal inflammatory response or infection after PPROM. AF and maternal serum samples were collected weekly after PPROM (23^+ 0^ − 34^+ 6^ gestational weeks) until delivery from twenty women and two women with possible chorioamnionitis with intact membranes. Levels of suPAR, PCT, interleukin-6 (IL-6), glucose, lactate dehydrogenase (LDH), and bacterial PCR were determined from AF and suPAR and PCT and IL-6 from maternal sera. Fetal infection or inflammation response were determined by the histology of the placenta after delivery. AF glucose was significantly lower and AF LDH higher in the fetal site histologic chorioamnionitis (HCA) group, while AF suPAR concentrations tended to be higher in this group. AF suPAR correlated significantly with AF glucose and LDH. Based on receiver operating characteristic (ROC) analysis, AF glucose had the best predictability for fetal site histological chorioamnionitis. The findings of AF PCT were insignificant considering HCA. AF glucose had the highest accuracy in predicting fetal site histologic chorioamnionitis. AF suPAR may be a promising marker; however, our findings were limited by a small study population.

**Supplementary Information:**

The online version contains supplementary material available at 10.1007/s43032-024-01678-6.

## Introduction

In developed countries, preterm delivery is the most common cause of neonatal death. Preterm premature rupture of membranes (PPROM) causes 30% of preterm deliveries. PPROM increases the risk of fetal infection or inflammatory response syndrome (FIRS). Both have adverse short- and long-term outcomes, such as an increased risk of neonatal sepsis, cerebral palsy (CP), neurodevelopmental disabilities, and bronchopulmonary dysplasia (BPD). Clinical chorioamnionitis with maternal symptoms– fever and tachycardia– is a late sign of intra-amniotic infection when delivery is indicated after PPROM [1, 2]. However, FIRS and fetal infection can appear earlier than the maternal symptoms of clinical chorioamnionitis. Biochemical markers of the amniotic fluid (AF) might help detect FIRS or fetal infection during the asymptomatic phase after PPROM [1–5] and could contribute to the delivery decision before worsening infection.

Serum soluble urokinase-type plasminogen activator receptors (suPAR) and procalcitonin (PCT) are novel biomarkers of immune activation used in adults and pediatric patients. In infectious diseases elevated suPAR concentrations are associated with development of sepsis, inflammatory response syndrome, and the risk of death in adults as well as in children. The findings for PCT in adult patients are similar [6–8]. Data on suPAR and PCT in AF after PPROM are scarce. As far as we are aware, in one previous study, AF PCT was not associated with histological chorioamnionitis [9]. In a recent single study, the median suPAR concentration in vaginally collected AF was highest in the group of FIRS accompanied with histological chorioamnionitis after PPROM [10].

Our study aimed to describe whether the concentrations of suPAR and PCT in AF or maternal sera could distinguish fetal site inflammatory response or infection from infection restricted to only the maternal site determined by placental histology after PPROM. Furthermore, we evaluated whether these novel biomarkers correlated with older biomarkers, such as interleukin-6 (IL-6), glucose, and lactate dehydrogenase (LDH), used to detect intra-amniotic FIRS or infection.

## Materials and methods

This prospective pilot study was performed at Tampere University Hospital (TAUH), Tampere, Finland, between 2015 and 2017. The study was approved by the TAUH Ethical Committee (Ethical code R15008) and was conducted according to the principles of the Declaration of Helsinki. Written informed consent was obtained from all participants.

The inclusion criteria included gestational weeks between 23^+ 0^ and 34^+ 6^ and PPROM or suspicion of intra-amniotic infection (IAI) without ruptured membranes in a singleton pregnancy. Gestational age was calculated according to the last menstrual period and corrected based on the first trimester ultrasonographic screening results. PPROM was defined as spontaneous rupture of membranes and diagnosed by a sterile speculum examination confirming pooling of amnion fluid and, if needed, using Actim Prom^®^ (Medix Biochemica, Kauniainen, Finland). The suspicion of IAI without PPROM was based on two of the following symptoms: uterine tenderness, maternal fever ≥ 38$$$$$$\:^\circ\:$$$$$$C, fetal tachycardia ≥ 160 beats/minute, maternal leukocytosis, or elevated CRP. Exclusion criteria included gestational weeks under 23^+ 0^ or above 34^+ 6^, fetal anomaly, or multifetal pregnancy.

At attendance, all women were tested by vaginal swap culture of Streptococcus agalactiae. Chlamydia and Neisseria gonorrhoeae were tested from urine samples using PCR, and urine bacterial culture was obtained. Cervical length and dilation were measured using transvaginal ultrasound and sometimes digital examination. Fetal position, estimated weight, umbilical artery pulsatile index, and amount of AF were determined using abdominal ultrasound.

All patients were admitted to a prenatal ward, and antibiotics (cefuroxime 1.5 g x 3 iv and azithromycin 500 mg x 1 per os) for 72 h were administered routinely. Corticosteroids (Celestone^®^ 12 mg twice 24 h apart) were given; tocolytic therapy was given during corticosteroid administration. Maternal blood white blood cell count (WBC) and serum C-reactive protein (CRP) were obtained daily during the three days of antibiotic treatment and every 2–3 days after. Fetal cardiotocography was recorded daily.

Transabdominal amniocentesis was performed under ultrasound guidance in the prenatal ward on the next day of admission by a senior consultant. Ten milliliters of AF were taken. AF samples for glucose and LDH were analyzed immediately, and the results were ready in three hours. The result of AF bacterial PCR was obtained in 72 h. AF samples and maternal serum for suPAR, IL-6, and PCT were taken but not analyzed for clinical use. AF and plasma samples were centrifuged and stored at -70◦C until analyzed. IL-6 level was determined using Quantikine^®^ HS ELISA or Quantikine^®^ ELISA (R&D Systems, Minneapolis, MN, US), suPAR level using suPARnostic^®^ AUTO Flex ELISA (ViroGates, Birker, Denmark), and PCT level using LIAISON^®^ BRAHMS PCT^®^ II GEN (DiaSorin, Saluggia, Italy) assay according to manufacturers’ instructions.

If there was no evidence for IAI based on AF samples (AF glucose ≤ 1 mmol/l and LD ≥ 280 U/l cut-offs were used) or clinical symptoms of chorioamnionitis or signs of fetal distress, pregnancy was continued, and the amniocentesis was repeated in a week with the same protocol. Otherwise, the decision to deliver was made.

**Fig. 1:**
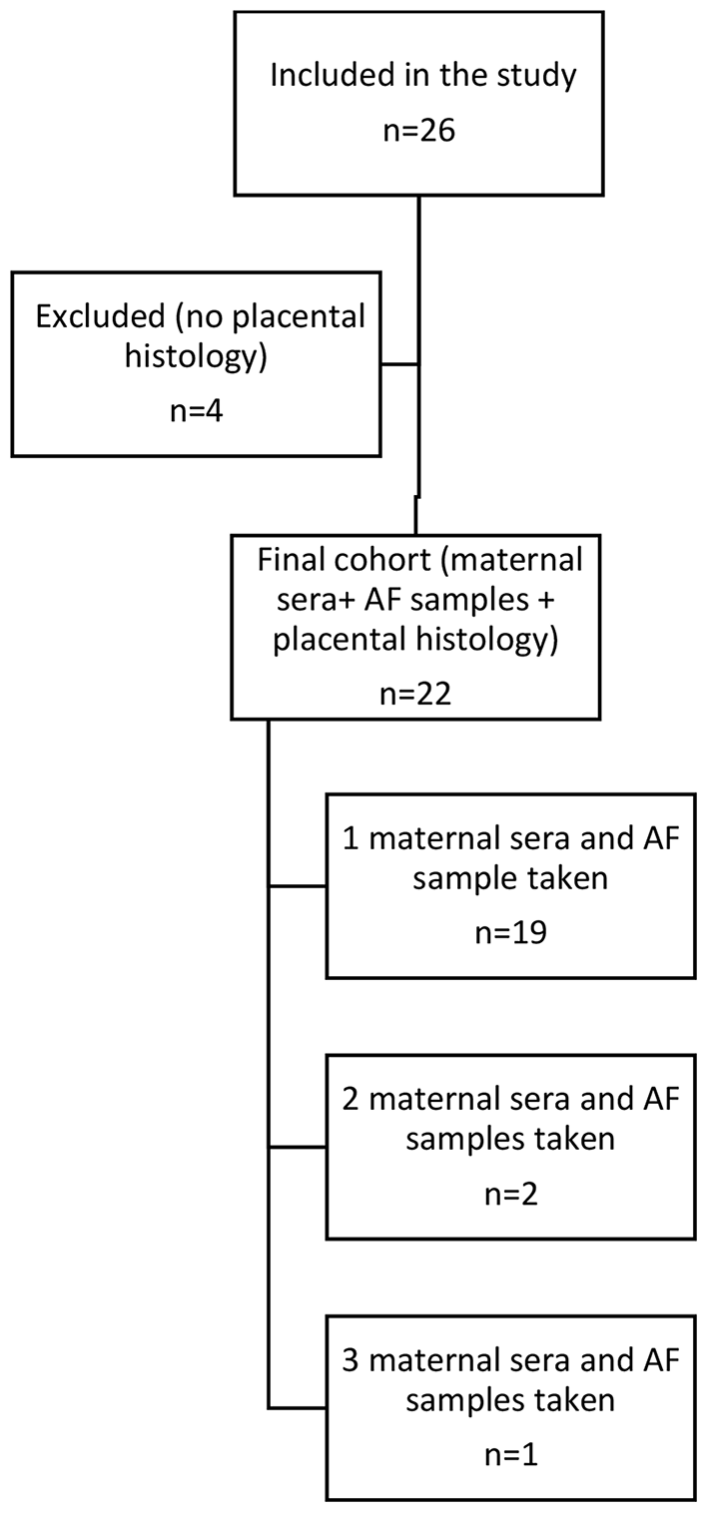
The patient flow diagram. The final cohort was formed from the sera and AF samples closest to delivery

Altogether, 26 women were included in the study, and 31 AF and blood samples were taken. One woman delivered in another hospital, and the histopathology of the placenta was not known. Furthermore, three placentas were mistakenly not sent for histopathological evaluation. These women (*n* = 4) were excluded; the final study population included 22 women. Most women had samples taken once, one woman had three amniocentesis, and three had two amniocentesis before delivery. The results closest to the delivery were taken for analysis. In addition, maternal sera samples closest to the delivery were chosen for the statistical analysis. Study population flow diagram is presented in Fig. 1. Umbilical cord samples for suPAR, PCT, and Il-6 were collected for further studies, and the results will be published separately. Clinical and laboratory data on women and newborns were collected from hospital medical records.

Histopathological evaluation was done using a pathology specialized in placental pathology. Placentas were evaluated, and histological chorioamnionitis on maternal or fetal sites was estimated using Redline’s classification and Amsterdam international consensus for placental lesions [11].

Statistical analysis was conducted using the Statistical Package for Social Sciences (SPSS) for Windows (IBM-SPSS version 23.0). The data are expressed as median and range. We compared continuous variables with the Mann–Whitney test and categorical variables with the Fisher exact or Chi-square test. The Spearman correlation test was used to calculate the correlation coefficients. Sensitivity and specificity were calculated. Receiver operating characteristic (ROC) curves were derived to evaluate the diagnostic performances of AF biomarkers in predicting fetal site histological chorioamnionitis; the area under the curve (AUC), with a 95% confidence interval (95% CI), was determined. We considered a p-value < 0.05 statistically significant.

## Results

### Study Population

The baseline characteristics of the women and neonates are presented in Table 1. The median gestational age at PPROM or suspected IAI was 27^+ 4^ (23^+ 0^- 34^+ 5^) gestational weeks. The median interval from attendance to study and delivery was three weeks. Twenty women had PPROM, and two suspected IAI without ruptured membranes. In a group suspected of IAI with intact membranes, one woman had perforated appendicitis and the other one had acute pancreatitis. They had the highest maternal CRPs (192 − 100 mg/mL) but no signs of histological chorioamnionitis. They also had the highest maternal PCT levels (0.780 − 0.3540 ng/L). A woman with appendicitis was operated after excluding IAI and delivered at term. A woman with pancreatitis caused by adenoma was operated and she delivered two weeks later. 14% of deliveries had spontaneous onset, 50% had an induction of labor for suspected IAI, and 36% had a cesarean section (CS) due to suspicious cardiotocogram (CTG) for fetal distress, partial placental abruption, breech presentation, or suspected IAI. The median birth weight was 1,380 g (range 630-3,265 g), and the median need for Neonatal Intensive Care Unit (NICU) was 23 days (range 0-105 days). None of the neonates had early-onset sepsis.

**Table 1:** Baseline characteristics of the study group

	*n* = 22
Age, years (mean, SD)	30(20–41)
BMI, kg/m^2^ (median, IQR)	26 (18–45)
Primiparas, %	54.5
ART, %	13,6
Previous preterm birth, %	18
CTG pathology, %	18
Induction of labor, %	50
GA at PPROM/suspected IAI (median, IQR)	27 + ^4^ (17 + ^0^-34 + ^5^)
GA at delivery (median, IQR)	30 + ^4^ (25 + ^0^-40 + ^3^)
Mode of delivery	
Vaginal delivery, %	64
Crash or emergengy cesarean section, %	18
Elective cesarean section, %	18
Birth weight, g (median, IQR)	1380 (630–3265)
Days in the NICU (median, IQR)	23 (0-105)

SD, standard deviation, BMI, body mass index, IQR, interquartile range, ART, assisted reproductive technology, CTG, cardiotocography, GA, gestational age PPROM, preterm premature rupture of membranes, IAI, intra-amniotic infection, NICU, neonatal intensive care unit

### Biochemical Markers and Histological Chorioamnionitis

**Fig. 2:**
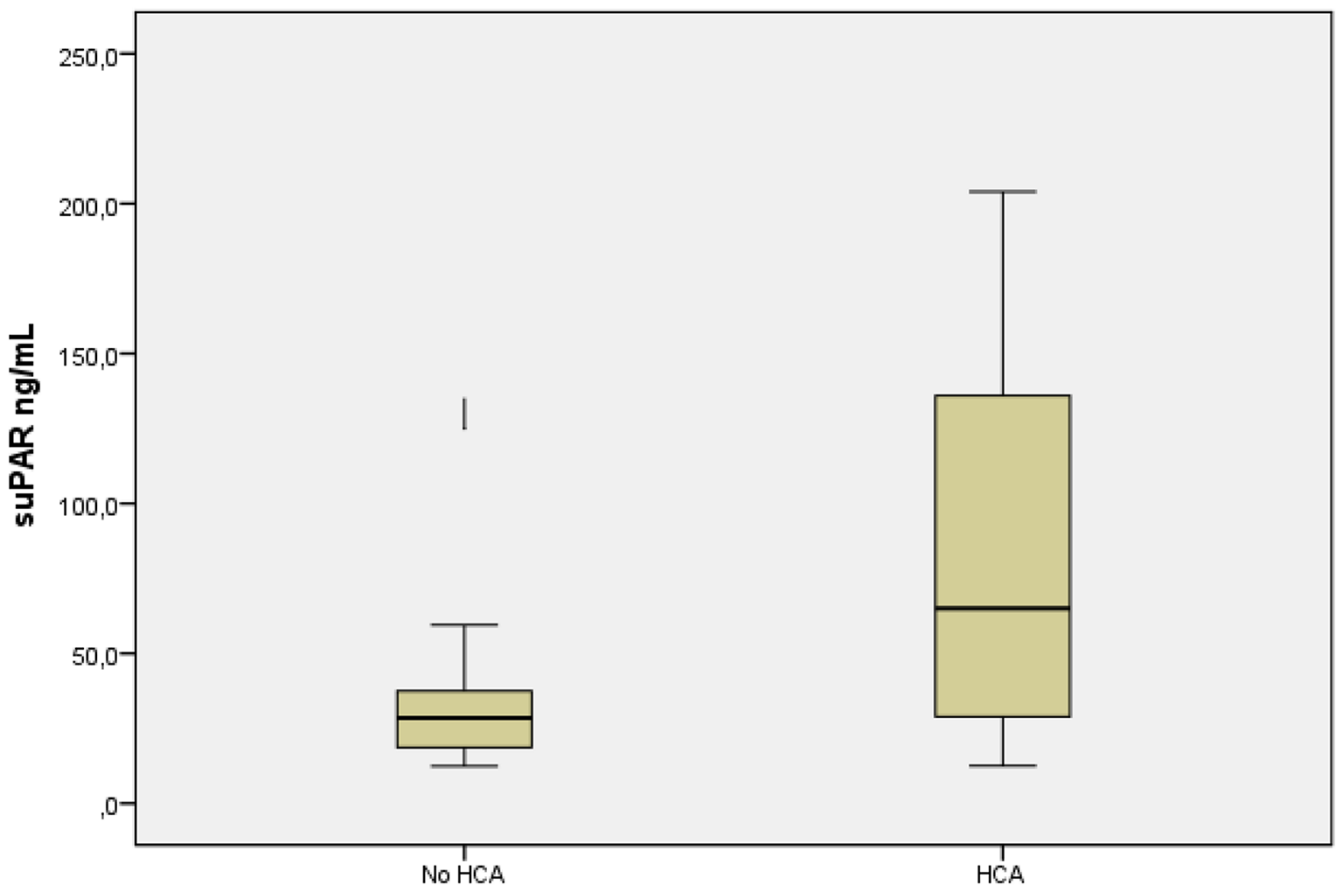
Amnion fluid suPAR levels. No HCA (*n*=9), HCA (*n*=13). The median concentrations of AF suPAR were lower in those without HCA than in those with HCA; 28,5 ng/mL (range from 12,4 to 59,5 ng/mL) vs. 58,0 ng/mL (range from 13,2 to 204,0 ng/mL), *p*=0,071. HCA; histological chorionamnionitis

Histological chorioamnionitis with fetal reaction was found in 59% (13/22) of women, and 41% (9/22) had histological infection restricted only to the maternal site or no infection (no-HCA group). The results of the biomarkers between the study groups are presented in Table 2. Biochemical markers from maternal sera were not significantly different between the study groups. There was a tendency for higher AF suPAR concentrations in the fetal site HCA group (median 58.0 ng/L) with a broader interquartile range than in the no-HCA group (28.5 ng/L), *p* = 0.071 (Fig. 2). No significant difference in AF PCT was found. The median AF glucose was significantly lower (0.5 mmol/L) in the HCA group than in the no-HCA group (1.9 mmol/L), *p* = 0.013. Furthermore, AF LDH was significantly higher in HCA (median 447 U/L) than in non-HCA (197 U/L), *p* = 0.049. IL-6 concentrations did not differ between the groups.

**Table 2:** Biochemical markers in maternal sera/plasma and in amniotic fluid

	HCA *n* = 13 median (IQR)	no HCA *n* = 9 median (IQR)	*p*-value
*Biochemical markers in* *maternal sera/plasma*			
SuPAR (ng/mL)	1,5 (0,9 − 2,10)	1,3 (1,0–3,1)	0,552
PCT (ng/L)	0,026 (0,0199-0,061)	0,032 (0,0199-0,354)	0,23
IL-6 (ng/L)	2,67 (0,68 − 24,95)	1,77 (0,47 − 26,33)	0,552
WBC (10^9^/L)	17,0 (4,5–24,0)	13,3 (8,0–20,0)	0,238
P-CRP (mg/L)	27 (1,0–63,0)	6,8 (3,7-192,0)	0,393
*Biochemical markers in* *amniotic fluid*			
SuPAR (ng/mL)	58,0 (13,2-204,0)	28,5 (12,4–59,5)	0,071
PCT (ng/L)	0,069 (0,027 − 0,156)	0,106 (0,035 − 0,214)	0,209
IL-6 (ng/L)	10,000 (455-19600)	1360 (81-10800)	0,110
Glucose (mmol/L)	0,5 (0,0–3,3)	1,9 (0,6 − 3,3)	0,013
LDH (U/L)	447(97- 4267)	197 (56–825)	0,049

IQR, interquartile range, SuPAR, soluble urokinase-type plasminogen activator receptor, PCT, procalcitonin, IL-6, interleukin 6, LDH, lactate dehydrogenase

### Predictive Performance of Biochemical Markers

AF glucose was the most accurate biomarker to distinguish between HCA and no-HCA based on the ROC graph and AUC analysis (Table 3). AUC resulted in 0.816 (95% Cl 0.631-1.000), *p* = 0.013. Bacterial PCR in the AF sample was positive in five women (four Ureaplasma urealyticum and one Capnocytophagus sputigena). All five women with positive bacterial PCR had AF glucose less than 0.5 mmol/L. When a cut-off value of 0.55 mmol/L was used, sensitivity was 62% and specificity was 100%. When considering AF LDH, suPAR, PCT, and IL-6 AUC remained poor. When a cut of value 32.5ng/ mL for suPAR was used, sensitivity (69%) was comparable to that of glucose; however, specificity was 67%. PCT had sensitivity and specificity remained under 50% and the best cut-off value was 0,087 ng/L.

**Table 3:** Area under the curve from ROC-analysis, sensitivity and specificity of biomarkers in amniotic fluid for histological fetal infection

Biochemical marker	AUC	95% CI	*p*-value	Cut-off	Sensitivity	Specificity
SuPAR (ng/mL)	0,709	0,450-0,968	0,193	32,50	69	67
PCT (ng/L)	0,354	0,068 − 0,623	0,336	0,087	46	44
IL-6 (ng/L)	0,709	0,450-0,968	0,193	3335	69	67
Glucose (mmol/L)	0,816	0,631-1,000	0,013	0,550	62	100
LD (IU/L)	0,667	0,419- 0,914	0,210	334,50	69	78

AUC, area under the curve, CI, confidential interval

### Correlations of Biochemical Markers

AF glucose had significant negative correlations between AF LDH, AF suPAR, and AF IL-6 (all *r* = 0.6, *p* = 0.01; 0.2; 0.04, respectively). AF LDH had a strong correlation with AF IL-6 (*r* = 0.8, *p* < 0.0001) and AF suPAR (*r* = 0.732, *p* < 0.0001). Furthermore, AF suPAR and AF IL-6 (*r* = 0.810, *p* < 0.0001) had a strong correlation. There was a tendency toward a positive correlation between maternal suPAR and maternal IL-6 in the HCA group (*r* = 0.6, *p* = 0.03); otherwise, no correlations were found in maternal biomarkers.

We also analyzed data excluding patients with appendicitis and pancreatitis. Infection and peritonitis with purulent excaudate in the abdominal cavity explained the high CRP and PCT. Otherwise, no significant differences were found between the study groups considering AF or maternal sera.

## Discussion

We explored whether two novel biomarkers– suPAR and PCT– could distinguish fetal site HCA from the maternal site or no HCA after PPROM and how these markers correlate with previously used markers such as glucose, LDH, and IL-6. In our study, AF glucose and LDH differed significantly between the HCA and no-HCA groups. Meanwhile, there was a tendency for higher AF suPAR in the fetal site HCA group. Furthermore, AF suPAR had a significant correlation with AF glucose and LDH. Low AF glucose concentration had the best predictability for fetal site HCA. Even if AF suPAR did not prove to be a more comparable biochemical marker than previously used, it might have some potential, and more extensive studies are needed.

Ruptured membranes are a risk factor for IAI [1, 2, 11]. It can be divided into clinical or subclinical, with the latter having less obvious clinical symptoms. The presence of IAI can be estimated by placental histology. Two types of HCA can be found: microbial invasion of the amniotic cavity (MIAC) and sterile inflammation reactions [1, 2]. Using Redline’s classification [11], histological infection or inflammation can be maternal or fetal. Both MIAC and sterile fetal inflammation are associated with increased neonatal morbidity and mortality, while infection restricted to the maternal site is less harmful to the fetus [3, 4, 8]. Since histological diagnosis of placenta comes after delivery, and clinical chorioamnionitis is a late sign of infection when delivery is recommended, it is important to explore new inflammatory markers from AF. During the asymptomatic phase after PPROM they could give further information about suspected FIRS or infection [12–15] and contribute to the delivery timing before worsening infection with a risk of fetal deterioration.

SuPAR is a soluble form of uPAR, a protein found in all cell membranes. Infection, inflammation, trauma, and other situations disturbing cell homeostasis cause uPAR to transform into a soluble form when it can be measured, for example, in plasma, liquor, and urine. SuPAR concentrations have been found to correlate positively with the immune system’s activation level [5–7]. In our study, AF suPAR concentrations were 20–40 times higher than concentrations of maternal sera. Previously, Uszynski et al. reported suPAR levels 100–200 times higher in AF than maternal sera in term pregnancies ending in a CS caused by complications like placental abruption during delivery [16]. In addition, we were able to measure it from all samples. We found a tendency to higher suPAR levels with a wider interquartile range in the fetal site HCA group. SuPAR correlated significantly with AF glucose and LDH, which were significantly different between the study groups. These findings may imply a possible association between suPAR and fetal HCA. This observation is in consistence with a recent larger study on vaginally collected AF suPAR. In this study the median suPAR concentration was significantly higher in the group FIRS (defined by high interleukin-6 level in the umbilical cord) with histological chorioamnionitis compared to the group neither FIRS nor HCA [10]. However, the neonates in our study were clinically in good condition, and no early-onset neonatal sepsis was found, which may have had an impact on our results. Based on ROC analysis, suPAR poorly predicted fetal site HCA. Our finding is restricted because of the small study size and clinically rather healthy neonates.

Procalcitonin is an acute-phase protein. It is a prohormone of calcitonin and is released mainly by neuroendocrine cells of the thyroid gland and bronchial K-cells of the lungs. In septic bacterial infections, almost all cells express PCT, which is shown to be an early sign of severe sepsis in adults [5]. Maternal PCT does not cross the placenta, and AF PCT is considered fetal in origin. Renal elimination of PCT is not a significant mechanism for PCT removal from plasma [17]. This could be one reason we did not find any differences in PCT between the study groups. PCT is useful in adult bacterial sepsis but shows no response to intracellular microorganisms, like mycoplasma, including Ureaplasma [18–21]. In the case of MIAC, the most common microbial finding in our study was Ureaplasma urealyticum, which may further explain the insignificant findings of PCT between the study groups. Our findings are consistent with previous studies on maternal sera and AF PCT after PPROM, where intra-amniotic infection was defined by either positive Gram stain or positive culture results [22] or placental histology [9].

Many biomarkers have been studied to distinguish fetal infection/inflammation reaction from maternal site reaction [8, 12, 15]. AF glucose is a widely used marker. IAI and AF glucose had an inverse correlation. AF glucose is independent of the amnion fluid index and reliable between the 5–95 percentiles [23]. AF glucose has been found to have an excellent sensitivity for identifying IAI in PPROM pregnancies. It cannot identify whether IAI is sterile or caused by microbial invasion; glucose levels are low in both cases. An AF glucose level of 0.56 mmol/L has been considered the optimal concentration for identifying IAI [24]. Our findings are consistent with these previous findings, and AF glucose had the best accuracy in distinguishing fetal HCA and no HCA.

LDH is a marker of acute inflammation and is measurable in different body fluids. It is part of the glycolytic pathway and catalyzes the reversible oxidation of lactate to pyruvate. LDH is a marker of apoptosis and reflects the damage of fetal membranes induced by bacteria or viral infection or mechanical damage, such as placental rupture [25, 26]. In our study, LDH was inferior to glucose in predicting fetal HCA and comparable to suPAR. Interleukin-6 is a proinflammatory cytokine expressed by T-cells and macrophages in response to infection or inflammation Elevated values have been found in cord blood in chorioamnionitis [14, 15]. We found AF IL-6 and glucose, as well as suPAR, to have a strong correlation, reflecting a possible association with fetal HCA. However, we found the predictability of IL-6 to fetal HCA to be poor.

Maternal sera samples of IL-6, PCT, and suPAR could not identify fetal HCA. A systematic review and meta-analysis of maternal inflammatory markers—CRP, IL-6, and PCT—in pregnancies complicated with PPROM and IAI had similar results [27]. After admission to the prenatal ward, all the women were given prophylactic antibiotics, which may have hindered maternal infection with raised laboratory values. Our findings also imply that HCA is clinically asymptomatic and not reflected in changes to maternal sera; markers in AF could be more sensitive to revealing HCA.

## Conclusions

In our study, AF glucose proved to be the best predictor of fetal HCA. AF suPAR may be a promising biomarker for identifying HC, but more extensive studies are needed. When considering PCT, our results confirm the insignificance of maternal sera or AF PCT in detecting IAI by the previous more extensive study.

## Electronic Supplementary Material

Below is the link to the electronic supplementary material.

Supplementary Material 1

## Abbreviations

AF: Amniotic fluid
BPD: Bronchopulmonary dysplasia
CP: Cerebral palsy
CRP: C-reactive protein
CS: Cesarean section
FIRS: Fetal inflammatory response syndrome
HCA: Histological chorioamnionitis
IAI: Intra-amniotic infection
IL-6: Interleukin 6
LDH: Lactate dehydrogenase
PCT: Procalcitonin
suPAR: Soluble urokinase-type plasminogen activator receptor
WBC: White blood cell count
