# Supplementary material for: Novel Biomarkers: Soluble Urokinase-Type Plasminogen Activator Receptor and Procalcitonin- and Histological Chorioamnionitis after Preterm Premature Rupture of Membranes
Source: Reprod Sci. 2024 Sep 3;31(10):3175–82. doi: 10.1007/s43032-024-01678-6 (PMC11438667; doi:10.1007/s43032-024-01678-6)
Supplement: Supplementary file 1 — Supplementary Material 1 [file 43032_2024_1678_MOESM1_ESM.pdf]

Elämän tähden

Pirkanmaan sairaanhoitopiiri/TAYS

PÄÄTÖSPÖYTÄKIRJA

17.6.2015

83 §
TIETEELLISEN TUTKIMUKSEN R15008 TOTEUTTAMINEN

Prof. Jukka Uotila on esittänyt tutkimuksen ETL-koodi R15008 toteuttamista toimialueella 4.

Päätös

Päätän, että

- tutkimus ETL-koodin R15008 voidaan toteuttaa "Sopimus tutkimuksen suorittamisesta ja lupa tutkimuksen toteuttamiseksi" - lomakkeen mukaisesti 31.12.2017 asti
- toimialueella 4
- Fimlab laboratoriot Oy:ssä
- sairaanhoidon palvelualueella

- tutkimuksen projektipäällikkönä toimii prof. Jukka Uotila naistentautien ja synnytysten vastuualueelta

- projektipäällikkö vastaa tutkimuksen kirjaamisesta tietojärjestelmiin ja laskutusten oikeellisuudesta sopimuksen mukaisesti.

- tutkimuksessa noudatetaan PSHP:n Tiedekeskuksen verkkosivuilla olevia ohjeita

Kari Nieminen
Toimialuejohtaja
Lasten ja naisten toimialue TA4

Päätösvallan perusteet ja sovelletut oikeusohjeet

Ohjekirje 42232/2014

Liitteet

Sopimus tutkimuksen suorittamisesta ja lupa tutkimuksen toteuttamiseksi 29.4.2015

Jakelu

Prof. Jukka Uotila, el Kati Jalkanen, tutkimuskoordinaattori Paula Vihtamäki (edelleen välitettäväksi Fimlab, SAPA)

Tiedoksi

Arkistotunnus: 430

Elämän tähden

Sopimus tutkimuksen suorittamisesta ja lupa tutkimuksen toteuttamiseksi

pvm: 29.4.2015
ETL-koodi: R15008

- [x] Tutkimus on käsitelty PSHP:n eettisessä toimikunnassa
- [ ] Tutkimuksesta on lausunto muualta, mistä ______
- [ ] Jatkoaikahakemus/muutos

(Täytetään vain muuttuneet kohdat tutkimuksen nimen ja tiedekoodin lisäksi)

1. Tutkimuksen yleistiedot

Tutkimuksen suomenkielinen nimi

Liukoinen urokinaasi-tyyppinen plasminogeenin aktivaattorin reseptori (SuPAR) pre-eklampsian, korionamnioniitin ja vastasyntyneen sepsiksen tunnistamisessa

- [ ] lääketutkimus — EudraCT-numero: ______
- [ ] laitetutkimus
- [x] Tutkijalähtöinen tutkimus
- [ ] Tilaustutkimus

Opinnäyte

- [x] väitöskirja
- [ ] pro gradu
- [ ] lisensiaattitutkimus
- [ ] syventävä työ
- [ ] muu, mikä: ______

2. Tutkijat Pirkanmaan sairaanhoitopiirissä

Suku- ja etunimi	Vastuualue/-yksikkö
Tutkija, joka vastaa tutkimuksesta (projektipäällikkö)	
Uotila Jukka vt prof., dos	Naistentautien ja synnytysten va
Muut tutkijat	
Jalkanen Kati, LL	Naistentautien ja synnytysten va
Tihtonen Kati	Naistentautien ja synnytysten va
Tammela Outi	Lastentautien va
Jalkanen Ville	Tehohoidon va
Aittoniemi Janne	Fimlab Oy
Tutkimushoitajat	

3. Tutkimuksen aikataulu Pirkanmaan sairaanhoitopiirissä

Alkamispäivämäärä: 15.1.2015 Päättymispäivämäärä: 31.12.2017

4. Tutkittavien lukumäärä

Tutkittavien henkilöiden lukumäärä PSHP:ssä:

60

5. Tutkimuspaikat (vastuualueet) Pirkanmaan sairaanhoitopiirissä

TA1:	☐ SIST	☐ KIHA		
TA2:	☐ KIRU	☐ GAST	☐ SYÖT	
TA3:	☐ TULES	☐ NIKU	☐ SKS	☐ SIKS
TA4:	☑ LAST	☑ NASY	☐ LAPS	
TA5:	☐ PSYK	☐ NUPS		
TA6:	☐ HOKA	☐ KUKA	☐ PUIS	☐ UUNI
TA7:	☐ PEA	☐ PPA		
SAPA:	☑ TEHO	☐ LEKA		
Fimlab laboratoriot Oy:		☑ FIMLAB		

Kuvantamiskeskus- ja apteekkiliikelaitos: ☐ SDIA ☐ KFI ☐ KNEF ☐ FYYSIKOT ☐ SAPT

Sydänkeskus Oy: ☐ SYDÄNKESKUS

Valkeakosken aluesairaala ☐

Vammalan aluesairaala ☐

Ylä-Pirkanmaan terveydenhuoltoalue ☐

Muu, mikä: ______

6. Raportointia varten tarvittavat tiedot

Tutkimuksen pääasiallinen tieteenala

- ☐ biolääketiede
- ☑ kliiniset lääketieteet
- ☐ ravitsemustiede
- ☐ kansanterveystiede
- ☐ hammaslääketiede
- ☐ liikuntatiede
- ☐ farmasia
- ☐ hoitotiede
- ☐ joku muu, mikä: ______

Tutkimuksen pääasiallinen luokka

- ☐ perustutkimus
- ☐ translationaalinen tutkimus
- ☑ kliininen tutkimus
- ☐ palvelujärjestelmää koskeva tutkimus, tarkennus:
  - ☐ terveyskeskusten toimintaa koskeva tutkimus
  - ☐ terveyspalvelujärjestelmän toimintaa koskeva tutkimus
  - ☐ tutkimus- ja hoitomenetelmien vaikuttavuutta koskeva tutkimus
- ☐ muu tutkimus, mikä ______

Tutkimuksen jaottelu

- ☐ terveydenhuollon yhteishanke

  PSHP:n ulkopuoliset tutkimuspaikat
  ______
  ______
  ______
  ______
  ______
- ☑ oman organisaation (PSHP) sisäinen hanke

7. Tutkimuksen rahoitussuunnitelma

Tutkimuksen rahoituslähteet

☐ vastuualueen EVO-raha ☐ kaupallinen tilaustutkimus, yritys: ______

☐ PSHP:n kilpailutettava EVO-raha ☐ muu, mikä: ______

Ulkopuolisen tutkimusrahoituksen hallinnointi

☐ PSHP/Tiedekeskus ☐ FinnMedi Oy ☐ Tampereen yliopisto ☐ Muu, mikä ______

Tutkimuksesta aiheutuvat kulut Pirkanmaan sairaanhoitopiirissä

Osastohoito, avohoito tai palveluyksikön tekemä tutkimus			Kustannukset							Kustannuksista vastaa		
kuvaus	tuotekoodi/kustannusluokka	suorittava vastuuyksikkö	hinta (€)	maksaja/maksulaji	asiakasmaksu (€)	asiakasmaksun maksaja	lkm/potilas	potilaiden määrä	yhteensä (€)	EVO-kustannuspaikka	x=ulkopuolinen rahoitus	hankekoodi
**Normaalia hoitoa**												
PVK, CRP		Fimlab						20	0			
LD lapsivedestä	4526		2				2	20	80			
bakteeri-pcr lapsivedestä							2	20	0			
glukoositaso lapsivedestä							2	20	0			
								yht.	80			
**Ylimääräiset hoidot, tutkimukset, materiaalit, palvelut, resurssit ja muut kulut**												
Aloitusmaksu		Fimlab	440				1	1	440			9R054
Ylläpito		Fimlab	165				2	1	330			9R054
Pre-analyyttiset työt: **-SUPAR lähete** -->äidin veri *		Fimlab	16				1	80	1280			9R054
Pre-analyyttiset työt: **-SUPAR lähete** -->äidin virtsa *		Fimlab	16				1	60	960			9R054
Pre-analyyttiset työt: **-SUPAR lähete** -->napaveri *		Fimlab	0				1	60	0			
Pre-analyyttiset työt: **-SUPAR lähete** -->lapsivesi *		Fimlab	16				2	20	640			9R054
*IL-6 äidin verestä	4842	Fimlab	54,25				2	20	2170			
*IL-6 lapsivedestä	4842	Finnlab	54,25				2	20	2170			

*prokalsitoniini äidin verestä	1773	Fimlab	46				2	20	1840			
*prokalsitoniini lapsivedestä	1773	Fimlab	46				2	20	1840			
*prokalsitoniini napaverestä	1773	Fimlab	46				2	20	1840			
näytteenotto		Fimlab	8				2	80	1280			9R054
								yht.	14790			

Laskutetaan laskutusaikana voimassaolevan hinnaston mukaan

Lisätietoja:
Laboratorio: SUPAR analytiikka tapahtuu tutkimusyhteistyönä Janne Aittoniemen kanssa, Fimlab
* Näytteet pakastetaan ja niiden määrittämisestä sovitään Fimlabin kanssa erikseen vuoden 2015 lopussa.

☐ P-asemalle perustettava tiedekansio
☐ Tutkijalle/ ☐ tutkimushoitajalle maksetaan erillistä korvausta

pvm: 29.4.2015
ETL-koodi: R15008

Tutkija, joka vastaa tutkimuksesta (projektipäällikkö)

Vakuutan, että projekti (ETL-koodi: R15008) vastaa syntyneistä kuluista,
niiltä osin kuin ne eivät kuulu potilaan normaaliin hoitoon ja
vakuutan perehtyneeni kliinisistä tutkimuksista annettuihin ohjeisiin ja määräyksiin PSHP:ssä ja noudattavani niitä.
Anon lupaa tutkimuksen toteuttamiseksi.

Päivämäärä: 2.6.2015

Allekirjoitus: [signature] Virka-asema: prof.
Nimenselvennys: Jukka Uotila Yhteystiedot: 03-31164485

Tutkimuksen yhteyshenkilö PSHP:ssä (jos eri kuin yllä):

Nimi: Virka-asema:
Yhteystiedot:

Hyväksyn tutkimussopimuksen rahoitussuunnitelman ja tutkimuksen toteutettavaksi yksikössäni

Toimi- /palvelualueen toimivaltaisen edustajan (hoitotieteen tutkimuksesssa lisäksi ylihoitajan) allekirjoitus

Päivämäärä:	Allekirjoitus:	Nimenselvennys ja toimi-/palvelualue, liikelaitos tms.
3.6.15	[signature]	Kirsi Kuismanen vaj NASY
10.6.15	[signature]	Merja Helminen vaj LAST
15.6.2015	[signature]	Terho Lehtimäki Fimlab Oy
17.6.2015	[signature]	Heli Leppikangas SAPA

Liitteet:
Eettisen toimikunnan myönteinen lausunto
Tutkimussuunnitelma tai sen lyhennelmä
Tiedote tutkittavalle
Tutkittavan suostumus

Tarvittaessa liitteeksi:
Sivutoimi-ilmoitus
Henkilökuntaan kuulumattoman tutkijan rekisteröinti-ilmoitus
Potilasasiakirjojen luovutus ja käyttölupahakemus/päätös
Rahoitussopimukset

Palautus:
Tutkimuskoordinaattorille

Jakelu
Tutkimuksesta vastaava tutkija (projektipäällikkö)
Toimi- ja palvelualueiden tutkimuskoordinaattorit
Tiedekeskus tarvittaessa
